# Supplementary material for: Early Detection of SARS-CoV-2 Omicron BA.4 and BA.5 in German Wastewater
Source: Viruses. 2022 Aug 25;14(9):1876. doi: 10.3390/v14091876 (PMC9503272; doi:10.3390/v14091876)
Supplement: Supplementary file 1 [file viruses-14-01876-s001.zip › viruses-1848629-supplementary.pdf]

viruses

MDPI

A)

B)

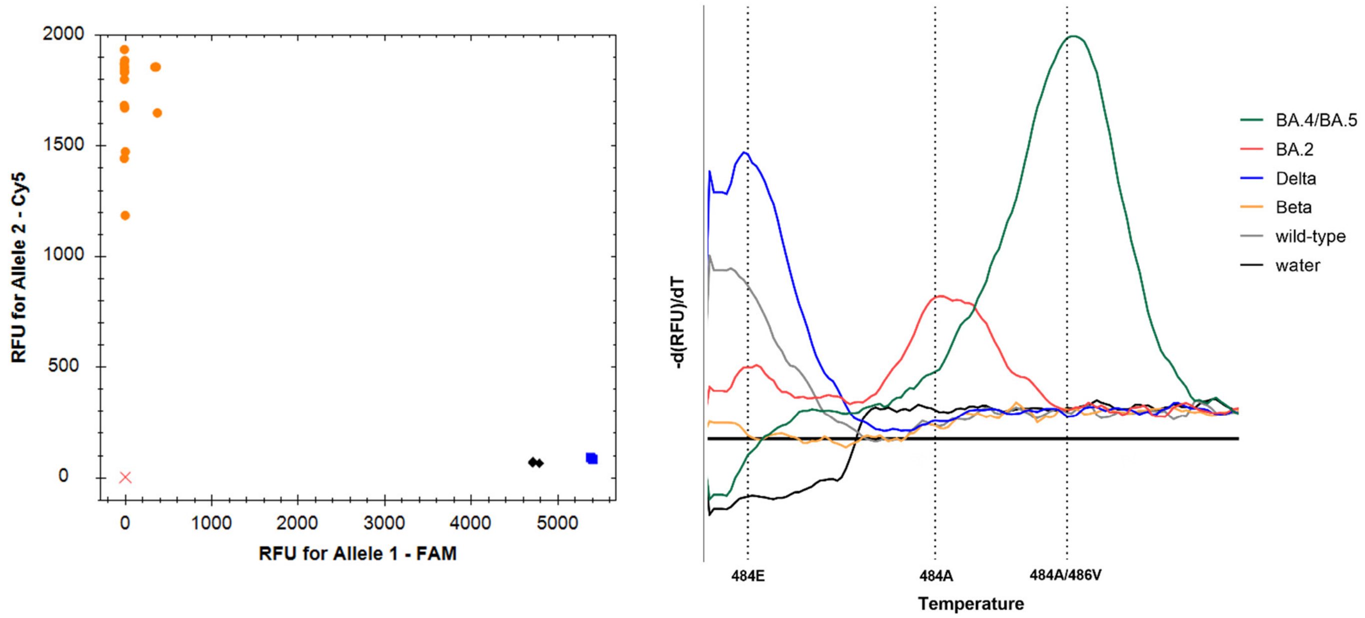

Figure S1. Specificity and sensitivity of PCR assays used in this study. **A)** Specificity and sensitivity of L452R assay. Samples spiked with inactivated SARS-CoV-2 variants and patient-derived swab samples were used for assay validation (n=3). SARS-CoV-2 VoCs Delta and BA.4/BA.5 were exclusively detected by the FAM-labelled probe detecting L452R (Delta shown in black and BA.4/BA.5 depicted in blue). Parental SARS-CoV-2, Alpha and Beta, were exclusively detected by the Cy5-labelled probe detecting L452 (all L452 carrying variants are depicted in orange). The red cross represents the water control. **B)** Specificity and sensitivity of variant-specific single nucleotide polymorphism PCR. Samples spiked with inactivated SARS-CoV-2 variants and patient-derived swab samples were used for assay validation.

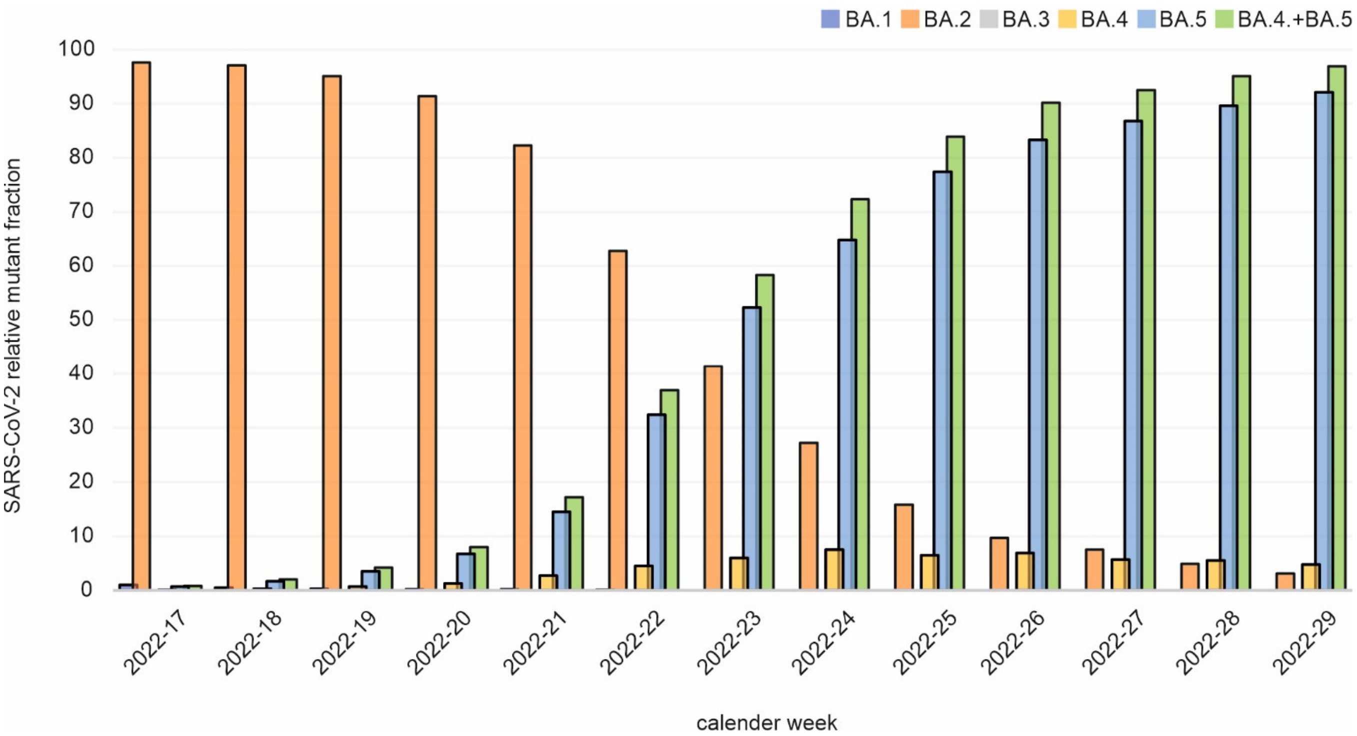

Figure S2. SARS-CoV-2 variant distribution in Germany based on public health data obtained from the official data repository of the federal Robert Koch Institute (RKI) in charge of national public health surveillance.
